# Experimental harvest regulations reveal that water availability during spring, not harvest, affects change in a waterfowl population

**DOI:** 10.1002/ece3.5743

**Published:** 2019-10-28

**Authors:** Benjamin S. Sedinger, Thomas V. Riecke, Christopher A. Nicolai, Russell Woolstenhulme, William G. Henry, Kelley M. Stewart

**Affiliations:** ^1^ College of Natural Resources University of Wisconsin – Stevens Point Stevens Point WI USA; ^2^ Program in Ecology, Evolution, and Conservation Biology University of Nevada Reno NV USA; ^3^ Department of Natural Resources and Environmental Science University of Nevada Reno NV USA; ^4^ Delta Waterfowl Foundation Bismarck ND USA; ^5^ Nevada Department of Wildlife Reno NV USA; ^6^ Nevada Waterfowl Association Fallon NV USA

**Keywords:** *Aix sponsa*, Bayesian, capture–mark–recapture, harvest dynamics, population estimation, wood duck

## Abstract

Population change is regulated by vital rates that are influenced by environmental conditions, demographic stochasticity, and, increasingly, anthropogenic effects. Habitat destruction and climate change threaten the future of many wildlife populations, and there are additional concerns regarding the effects of harvest rates on demographic components of harvested organisms. Further, many population managers strictly manage harvest of wild organisms to mediate population trends of these populations. The goal of our study was to decouple harvest and environmental variability in a closely monitored population of wild ducks in North America, where we experimentally regulated harvest independently of environmental variation over a period of 4 years. We used 9 years of capture–mark–recapture data to estimate breeding population size during the spring for a population of wood ducks in Nevada. We then assessed the effect of one environmental variable and harvest pressure on annual changes in the breeding population size. Climatic conditions influencing water availability were strongly positively related to population growth rates of wood ducks in our study system. In contrast, harvest regulations and harvest rates did not affect population growth rates. We suggest efforts to conserve waterfowl should focus on the effects of habitat loss in breeding areas and climate change, which will likely affect precipitation regimes in the future. We demonstrate the utility of capture–mark–recapture methods to estimate abundance of species which are difficult to survey and test the impacts of anthropogenic harvest and climate on populations. Finally, our results continue to add to the importance of experimentation in applied conservation biology, where we believe that continued experiments on nonthreatened species will be critically important as researchers attempt to understand how to quantify and mitigate direct anthropogenic impacts in a changing world.

## Graphical abstract

We experimentally manipulated harvest regulations to decouple harvest regulations from density dependent effects and used 9 years of intensive capture–mark–reencounter data to estimate annual wood duck abundance and band recovery rate. We examined how harvest and water availability affect change in breeding abundance. We found a positive relationship between annual water availability and change in breeding abundance but no relationship between harvest rate and change in breeding abundance.

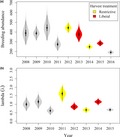

## 1 INTRODUCTION

A fundamental goal of applied ecology is to understand the mechanisms governing the dynamics of populations. The difference between the number of entries (births and immigration) into and exits (deaths and emigration) from a population over determines temporal change in abundance (Pradel, 1996; Williams, Nichols, & Conroy, 2002). Vital rates, like survival, may be affected by environmental conditions (Amundson & Arnold, 2011; Aubry et al., 2013), anthropogenic factors (Arnold & Zink, 2011; Loss, Will, & Marra, 2012), and demographic stochasticity (Lande, 1993). Vital rates are also influenced by a population's evolutionary history (Koons, Pavard, Baudisch, & Metcalf, 2009; Stearns, 1992). The demographic buffering hypothesis posits that evolutionary history can reduce variability in vital rates that, proportional to other vial rates, have the greatest potential to influence change in the population growth rate (*λ*) over the long term (Boyce, Haridas, & Lee, 2006; Koons, Gunnarsson, Schmutz, & Rotella, 2014). This is because, all else being equal, *λ* declines as its temporal variance increases so selection should favor reduced variability in these vital rates through time (Gillespie, 1977; Koons et al., 2014). In this way, annual variation in *λ* might be more influenced by vital rates that fluctuate with changing environments. For example, waterfowl populations, with the exception of teal, are more sensitive to changes in adult survival rate than fecundity (Koons et al., 2014), but much of the annual variation in these populations is driven by variation in fecundity (Hoekman, Mills, Howerter, Devries, & Ball, 2002; Raveling & Heitmeyer, 1989; Sedinger et al., 2016). In this case, changes in survival rate can have important effects on population dynamics; if hunting increases total annual mortality, then hunting might lead to population‐level declines in abundance (e.g., black ducks, *Anas rubripes*, Conroy, Miller, & Hines, 2002).

Successive years of declining abundance, from anthropogenic disturbance or other causes, can initiate management actions that are directed at specific vital rates to induce population‐level change in abundance (Loss, Will, & Marra, 2012). The most common example is the use of take regulations to regulate annual survival (Runge & Boomer, 2005). Population managers have suggested liberal take regulations as a method of reducing overabundant (Alisauskas et al., 2011) or exotic invasive (Bomford & O'Brien, 1995) populations and restrictive take regulations as a method of increasing populations that are declining (Runge & Boomer, 2005). These conservation actions can be inefficient, however, if they are directed at vital rates that are not affecting declines (Rice, Haukos, Dubovsky, & Runge, 2010; Richkus, 2002), if the vital rate in question is relatively inflexible because of demographic buffering (Boyce et al., 2006; Koons et al., 2009), or if directed at vital rates that are affected by density‐dependent feedbacks (Amundson & Arnold, 2011; Sedinger & Herzog, 2012), which are common among vertebrate populations (Sibly & Hone, 2002; Williams et al., 2002), including waterfowl (Gunnarsson et al., 2013; Koons et al., 2014).

Harvest is deliberate take, and harvest regulations are used as a tool to manage potential harvest effects on survival rates at the population level; if harvest adds to natural sources of mortality, then restrictive regulations, that reduce the harvest rate, should induce population‐level increases in survival rate (Anderson & Burnham, 1976), and consequently, abundance, assuming no change in recruitment. However, if harvest mortality and natural mortality are both affected by population density and if harvest mortality compensates for natural mortality, then restrictive harvest regulations will be largely ineffective at increasing population‐level survival rates (Anderson & Burnham, 1976; Sedinger & Herzog, 2012). Additionally, if the trajectory of a population is governed primarily by fecundity, management actions aimed at influencing survival may be ineffective. Therefore, it is important to understand what influences variability in specific vital rates, how variability in vital rates affects variability in abundance and, ultimately, the specific demographic factors affecting changes in population size. However, many longitudinal datasets that are currently available to assess the effects of harvest on population vital rates, like survival, are inherently flawed because harvest regulations are confounded with population size, which also can affect vital rates through processes like negative density dependence (Sedinger & Herzog, 2012).

Abundance is commonly estimated from surveys that count individuals (Lancia, Kendall, Pollock, & Nichols, 2005). In the United States, many large‐mammal harvest programs determine annual regulations from counts of adults and juveniles, from which age and sex ratios are calculated in addition to population size. These data are then used to establish harvest quotas (Bishop, White, Freddy, & Watkins, 2005; McCullough, 1994), in an effort to affect population trajectories. Waterfowl are surveyed during the breeding season in the United States and Canada to determine the number of breeding pairs, which informs population and harvest management (U.S. Fish & Wildlife Service report, 2017). While abundance surveys are widely used to inform research and management, they also have limitations associated with the estimation of detection probability (Graham & Bell, 1989; Pagano & Arnold, 2009; Pollock & Kendall, 1987; Samuel & Pollock, 1981; Zimmerman, Sauer, Fleming, Link, & Garrettson, 2015). Weather and visibility during surveys affects both the ability of observers and the behavior of organisms being surveyed (Caughley & Goddard, 1972). Further, variation in the timing of surveys from year to year may create variation in the distribution of surveyed animals (especially for multispecies surveys, e.g., waterfowl breeding population surveys, avian breeding bird surveys) with respect to the area sampled (Ross, Hooten, DeVink, & Koons, 2015). Given existing concerns with the efficacy and cost of aerial survey approaches, capture–mark–recapture (CMR) approaches have increasingly been used as an alternative for estimating abundance of waterfowl in North America (Alisauskas et al., 2011; Alisauskas, Arnold, Leafloor, Otis, & Sedinger, 2014). Typically, CMR estimates of population abundance differ substantially from aerial surveys, where the abundance of extremely abundant or cryptic species is often underestimated using traditional survey approaches (Alisauskas et al., 2014).

To fully assess the relationships between harvest and population trends of wild organisms, ecologists require experiments where potential factors influencing demography vary independently, while accurately estimating population size and harvest rates. In this manuscript, we use an experimental approach to harvest regulation for a population of wood ducks (*Aix sponsa*) in western Nevada to isolate some of the factors that are thought to influence annual population change. We focus on the effects of water availability during spring and harvest, as water availability has been shown to influence population dynamics of waterfowl (Raveling & Heitmeyer, 1989), while harvest effects on waterfowl population dynamics are much less clear (Anderson & Burnham, 1976; Cooch, Guillemain, Boomer, Lebreton, & Nichols, 2014; Sedinger & Herzog, 2012). Our primary objective was to assess the influence of harvest and water availability during the breeding season on annual variation in population size of wood ducks. Critically, we use capture–recapture methods to estimate breeding abundance and harvest effects on this cryptic, difficult to survey species. We hypothesize that population size is primarily affected by recruitment and factors that influence recruitment, like water availability during the breeding season. Thus, we predict that there will be greater population growth during high water years, relative to low water years. Further, we hypothesize that factors influencing the survival process, like harvest, will have little influence on change in breeding abundance and therefore predict that harvest rates will not influence change in abundance from 1 year to the next.

## 2 MATERIALS AND METHODS

### 2.1 Study organism

**Figure 1:**
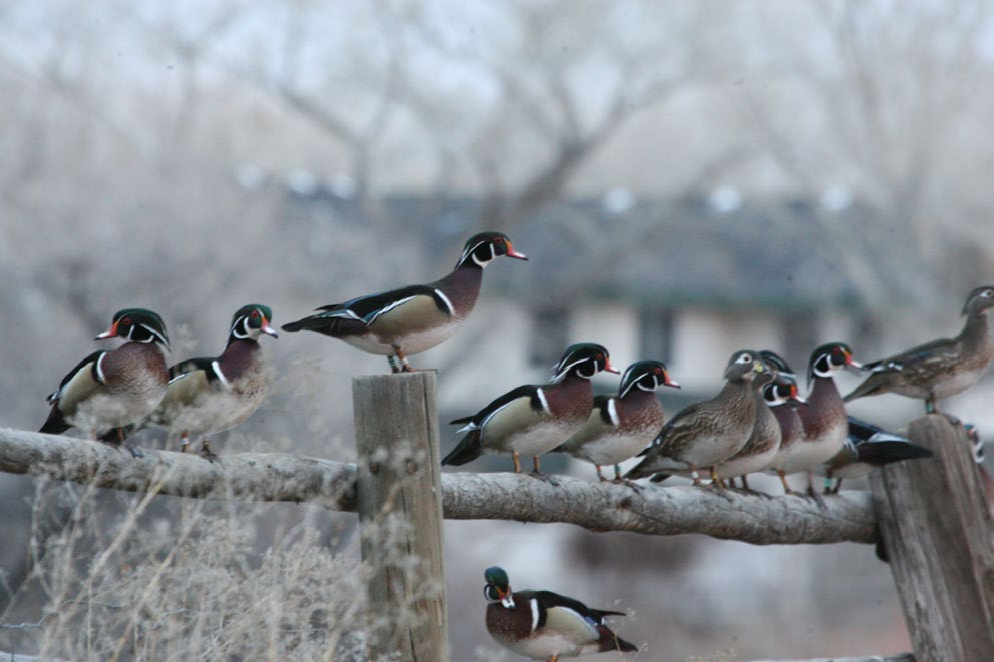
A group of wood ducks (*Aix sponsa*) in Nevada. Photograph credit: Chris Nicolai

Wood ducks (Figure 1) are a perching duck species that nest in tree cavities. Individuals begin breeding during their second year of life. They are widely distributed across much of the United States and southern Canada and are commonly hunted during autumn and winter months (Bellrose & Holm, 1994).

### 2.2 Data collection

**Figure 2:**
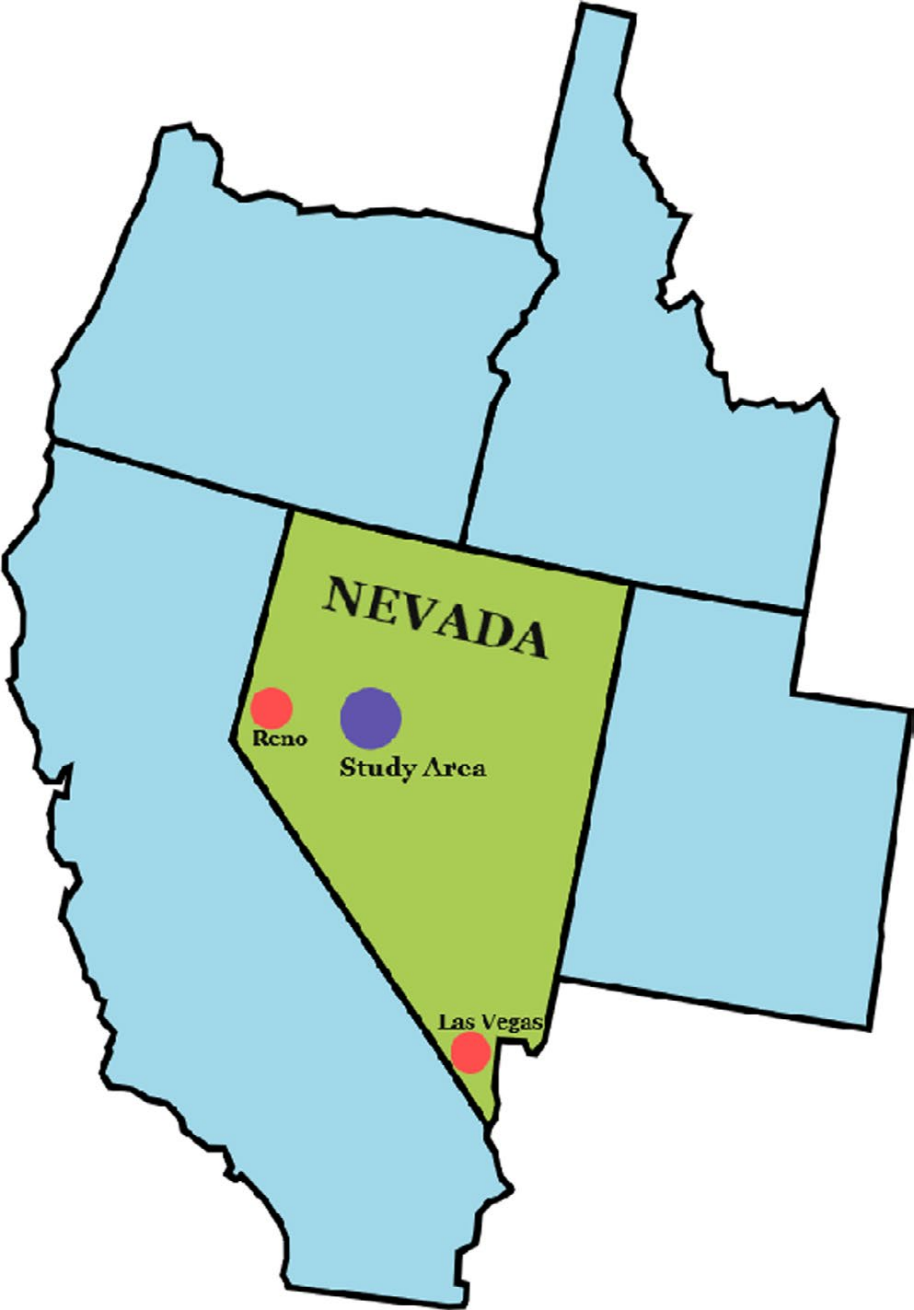
A map of the state of Nevada indicating the location of the long‐term demographic project in Churchill County, Nevada

Data were collected from March 2008 to September 2016 as part of a long‐term demographic study of wood ducks that reside along the Carson River near the town of Fallon in Churchill County, Nevada (39.4749°N, 118.7770°W; Figure 2). Average rainfall in the study area is 4.4 cm during the breeding season (April–July; Fallon, NV Weather Station). Available habitat for wood ducks in the study area is confined to the Carson River corridor and nearby agricultural irrigation ditches. River flows correlate with irrigation demands from agricultural producers and with the annual snowpack, which determines water availability during the breeding season.

We encountered wood ducks early in the breeding season during March and April at bait sites using rocket nets and air cannons (Dill & Thornsberry, 1950). During initial capture, we banded all individuals with uniquely coded USGS aluminum bands and uniquely coded plastic tarsal bands that could be read with a spotting scope from a distance. We determined age and sex using plumage characteristics (Carney, 1992) and transitioned juveniles to adults on July 1st the year after hatching; during spring months, individuals were classified as being second year (SY) or after‐second‐year (ASY). In addition to physical captures, we encountered individuals by resighting tarsal bands with spotting scopes because a high proportion of the population (>75%) is marked. We also attempted to capture all females incubating eggs in nest boxes (*n* ~420) in the study area. Finally, hunters harvested and reported marked individuals during the hunting season (October–January), and we retrieved these data from the USGS Bird Banding Laboratory. We downloaded snowpack data, for the month of April when snowpack is greatest, from the NRCS SNOTEL network for the headwaters of the Carson River in the Sierra Nevada mountain range. All methods and procedures involving handling wood ducks were approved by the Institutional Animal Care and Use Committee, Protocol #403, at the University of Nevada, Reno. All wood ducks in the study were banded under Federal bird banding permit #23713.

### 2.3 Harvest experiment

We experimentally manipulated daily bag limit for wood ducks to disentangle density‐dependent processes from the process of setting harvest regulations. Prior to the experiment, hunters were permitted to harvest seven wood ducks per day, which are the maximum allowed under federal harvest frameworks in the Pacific Flyway. In 2011 and 2013, we restricted harvest by reducing the daily bag limit from seven wood ducks per day to one wood duck per day (“restrictive treatment year”).

### 2.4 Analysis: Bayesian closed‐capture estimates of abundance

We analyzed seasonal capture–mark–recapture data (CMR) using two‐sample closed‐population models (Lincoln, 1930; Otis, Burnham, White, & Anderson, 1978) in a Bayesian framework (Kéry & Schaub, 2011) with data augmentation (Tanner & Wong, 1987). This allowed us to estimate spring breeding population size or the total number of second year (SY) and after‐second‐year (ASY) female wood ducks in the study area at the beginning of the breeding season, which we defined as March and April (Sedinger, Stewart, & Nicolai, 2018). This class of models assumes closure during sampling, for example, no immigration or emigration, because these types of movement will bias estimates of abundance. We assumed closure, as approximately 85% of direct and indirect band recoveries occur within the study area, and our sampling window was limited to 2 months. We estimated annual (*t*) population size (*N_t_*) during spring, where we first estimated each individual's (*i*) latent state (*z_i,t_*) given secondary occasion specific (*j*) capture–recapture data (*y_i,t,j_*) and inclusion ($\Omega_{t}$) and detection (*p_t_*) probabilities.

$$\begin{array}{c} z_{i,t}∼\text{Bernouli}\left(\Omega_{t}\right), \end{array}$$

(1) $$\begin{array}{c} y_{i,t,j}∼\left\{\begin{array}{c} \text{Bernouli}\left(p_{t}\right),\;\text{if}\;z_{i,t}=1\\ 0,\;\text{if}\;z_{i,t}=0, \end{array}\right. \end{array}$$

$$\begin{array}{c} N_{t}=\sum_{i=1}^{t}z_{i,t} \end{array}$$

We used vague priors for inclusion and detection parameters,

(2) $$\begin{array}{c} \Omega_{t}∼\text{Uniform}\left(0,1\right),\\ p_{t}∼\text{Uniform}\left(0,1\right). \end{array}$$

We augmented observed capture histories with 1,500 all‐zero capture histories, which is greater than the total number of wood ducks thought to inhabit the study area. We ran three chains for 20,000 iterations, thinned by two and discarded the first 3,000 iterations as burn‐in. Finally, we visually checked chains for convergence, and used the Brooks–Gelman–Rubin statistic ($\hat{R}$) <1.1 as an assessment of convergence (Brooks & Gelman, 1998). We derived the population growth rate (*λ*) for the breeding population as follows:

(3) $$\begin{array}{c} \lambda_{t}=\frac{{\hat{N}}_{t+1}}{{\hat{N}}_{t}}. \end{array}$$

We conducted analyses in JAGS (Plummer, 2003) using the “jagsUI” package (Kellner, 2016) in R 3.6.1 (R Core Team, 2019).

### 2.5 Analysis: Bayesian linear regression models

We then used Bayesian linear regression models to examine how population growth rate (*λ*) is influenced by direct recovery rates (ƒ) which are an index of harvest (Anderson & Burnham, 1976) and current amounts of snowpack (*γ*) at the headwaters of the Carson River in the Sierra Nevada Mountains,

(4) $$\begin{array}{c} \lambda_{t}=\alpha +\beta_{f}*f_{t}+\beta_{\gamma }*\gamma_{t}+\varepsilon_{t},\\ \varepsilon_{i}∼\text{Normal}\left(0,\sigma^{2}\right), \end{array}$$

We used snowpack at the headwaters of the Carson River, during the month of April, as a measure of environmental conditions during breeding because all water in the Carson River system originates in the Sierra Nevada Mountains, and flows are regulated by the annual snowpack. We estimated direct recovery rates as the quotient of total hunter recovered banded individuals in year *t* and total preseason (July–September) encounters of banded individuals in year *t*. All covariates were *z*‐standardized (*µ* = 0, *SD* = 1) before analysis.

We used vague priors for intercept (*α*), slopes (*β*), and error (*σ*) terms,

(5) $$\begin{array}{c} \alpha ∼\text{Normal}\left(0,0.01\right),\\ \beta ∼\text{Normal}\left(0,0.01\right),\\ \sigma ∼\text{Uniform}\left(0,10\right). \end{array}$$

We ran three chains for 20,000 iterations and discarded the first 3,000 iterations as burn‐in. We visually checked chains for convergence and used the Brooks–Gelman–Rubin statistic ($\hat{R}$) <1.1 as an assessment of convergence (Brooks & Gelman, 1998). We used Bayesian *p*‐values to assess model fit, where values near .5 indicate adequate fit (Gelman, 2013; Kéry, 2010). Finally, we assessed the strength of covariate effects by computing 95% Bayesian credible intervals (BCI) and *f*‐values that describe the proportion of posterior distributions that share the same sign as the beta coefficient (Plummer, 2003). As such, *f*‐values provide a measure of the probability that a covariate either had an effect or did not have an effect on the response variable. We conducted analyses in JAGS (Plummer, 2003) using the “jagsUI” package (Kellner, 2016) in R 3.3.2 (R Core Team, 2019).

## 3 RESULTS

**Figure 3:**
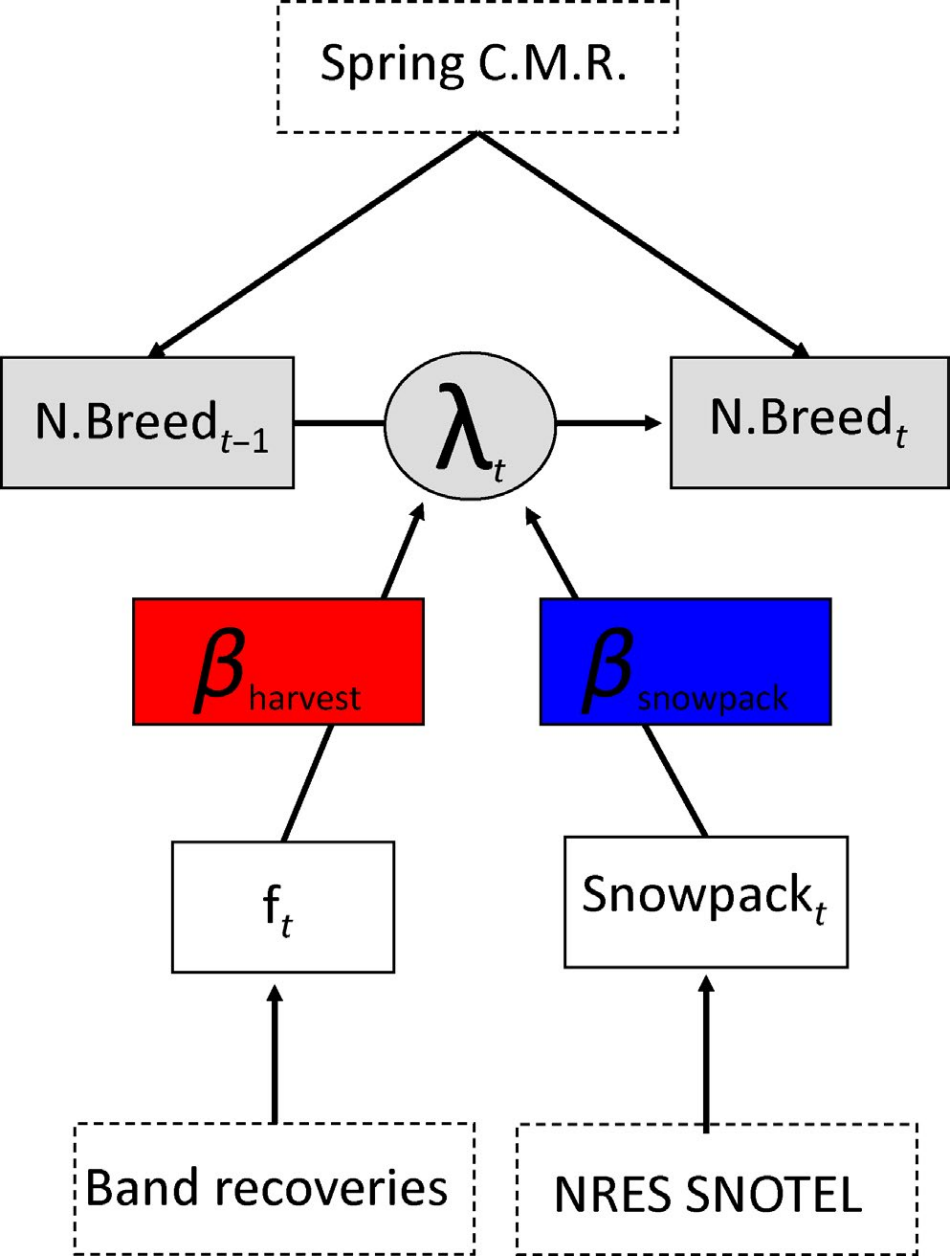
A directed acyclic graph of the capture–recapture model used to estimate drivers of population growth rates of wood ducks marked in Churchill County, Nevada (2008–2016). Data are represented by dashed boxes. Estimated parameters are represented by circles (population growth rate) and solid boxes (associated variables) in the analysis and how they relate (colored solid boxes) to population growth rate (circle)

Between 2008 and 2016, we encountered 2,565 unique individuals during March and April. Detection probability varied across years from *p*_2008_ = .23 (95% BCI: 0.17–0.28) to *p*_2014_ = .567 (95% BCI: 0.517–0.616). Estimates of breeding population size ranged from *N*_2016_ = 382 (95% BCI: 355–416) to *N*_2010_ = 893 (95% BCI: 736–1,117; Figure 3a). Detailed results from this analysis are outlined in a table in the supplementary material (Appendix S1).

**Figure 4:**
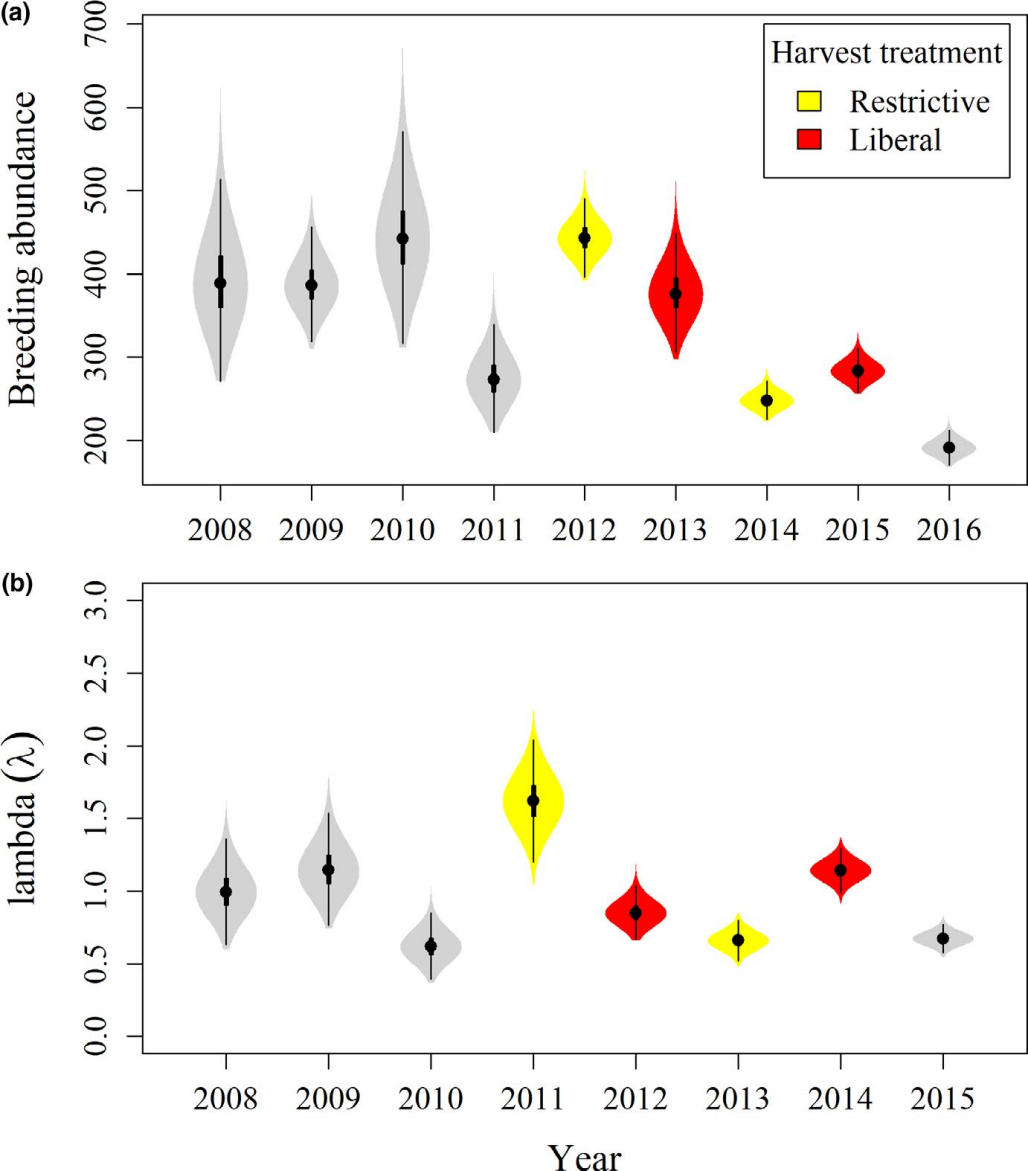
Wood duck population estimates of (a) total abundance during breeding season, and (b) population growth rate (*λ*); from Bayesian closed‐capture analysis of capture–mark–reencounter data from Churchill County, Nevada (2008–2016)

Estimates of the population growth rate ranged from *λ*_2010–2011_ = 0.623 (95% BCI: 0.468–0.802) to *λ*_2011–2012_ = 1.622 (95% BCI: 1.331–1.926; Figure 3b). From the regression analysis, population growth rate was positively affected by spring snowpack (*β* = 0.257, 95% BCI: −0.047 to 0.567, *f* = 0.961), but not by direct recovery rate which ranged from 0.049 to 0.17 during the study (*β* = −0.001, 95% BCI: −0.311 to 0.314, *f* = 0.501; Figure 4). Bayesian *p*‐values indicate good model fit (*p*‐value = .578). Detailed results from this analysis are outlined in a table in the supplementary material (Appendix S2).

## 4 DISCUSSION

**Figure 5:**
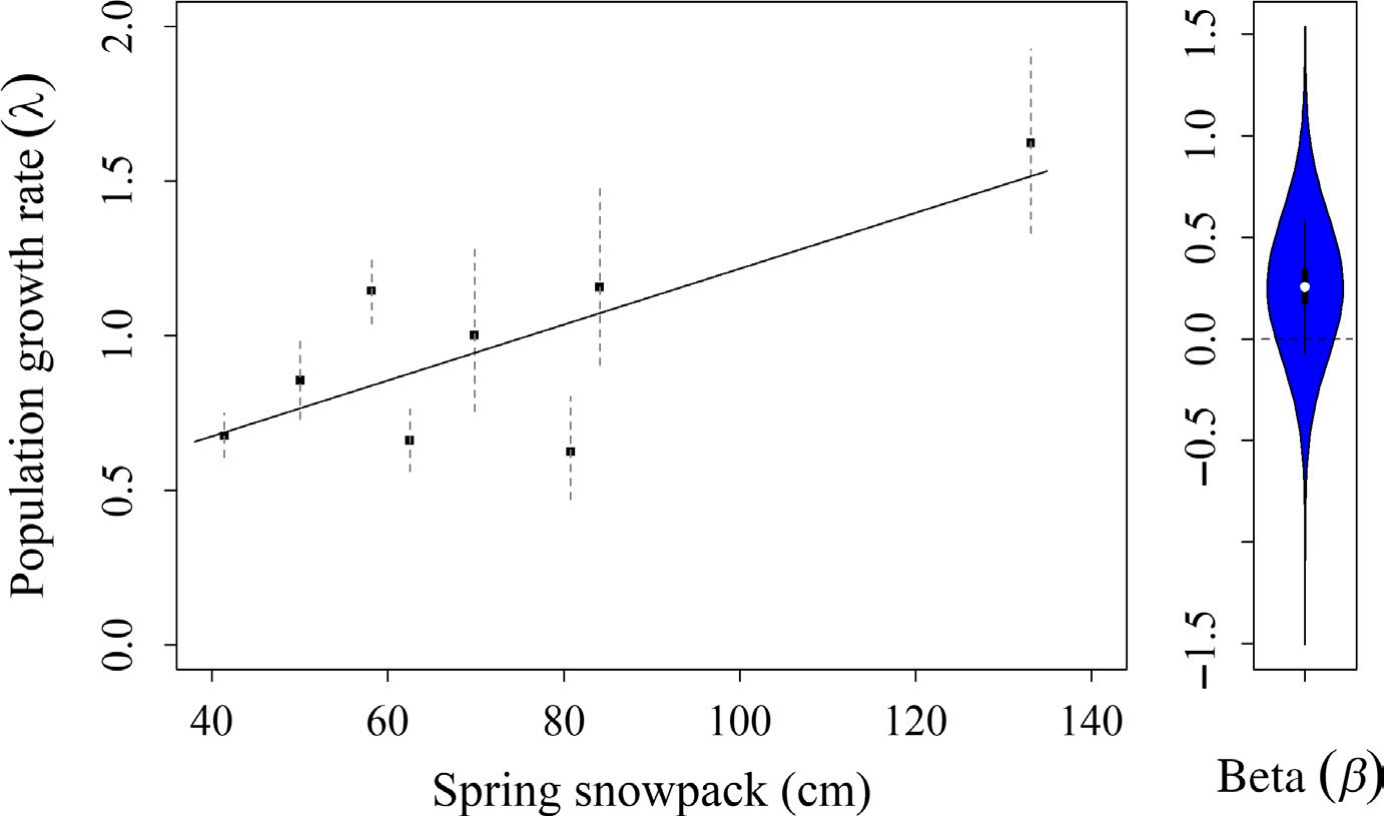
Relationship between the population growth rate (*λ*) and snowpack during spring at the headwaters of the study area from wood duck capture–mark–recapture study in Churchill county, Nevada (2008–2016). Beta represents the slope coefficient from regressions analysis and is positive (*β* = 0.257, 95% BCI: −0.047 to 0.567, *f* = 0.961)

Our results indicate that population trajectories of wood ducks in western Nevada are strongly positively affected by current year spring snowpack in the Sierra Nevada mountains (Figure 5). In this system, snowpack determines water availability during the summer when females are nesting and caring for broods. Thus, it provides an index of water availability and environmental conditions during the breeding season. In this way, the wood duck population we studied is reliant on the amount of water available during the breeding season to sustain itself. All waterfowl are closely tied to wetland habitats throughout their life cycles (Baldassarre & Bolen, 1994), and increased precipitation is commonly associated with increased productivity in waterfowl populations (Raveling & Heitmeyer, 1989). Wet years provide increased cover for ground‐nesting birds, which often correlates with nest success (Winter, Johnson, & Shaffer, 2005), but wood ducks nest in tree cavities, where more water on the breeding grounds is likely not affecting the quality of available nesting sites in the same way. Water might affect physiological condition of breeding females if food resources increase during wet years (Fleskes, Yee, Yarris, & Loughman, 2016). Further, body condition can affect breeding propensity (Warren et al., 2014), clutch size (Ankney, Afton, & Alisauskas, 1991), and, consequently, recruitment. Habitat conditions also affect duckling survival through food availability (Cox et al., 1998) or predation‐related effects (Pieron & Rohwer, 2010). Consequently, available water may positively influence several components of waterfowl recruitment and survival processes.

Waterfowl population surveys were among the first large‐scale monitoring programs for wildlife in North America and have provided valuable information about changes in waterfowl abundance for the last 60 years (U.S. Fish & Wildlife Service, 2017). From these surveys, we can detect three large declines in total duck abundance over the last 60 years, the first in the 1960s, the second in the 1980s, and the third in the early 2000s. In agreement with our study, these trends are highly correlated with indices of breeding habitat conditions (e.g., “May ponds”), but there have also been concerns about the effects of hunting on waterfowl populations through time (Conroy et al., 2002).

**Figure 6:**
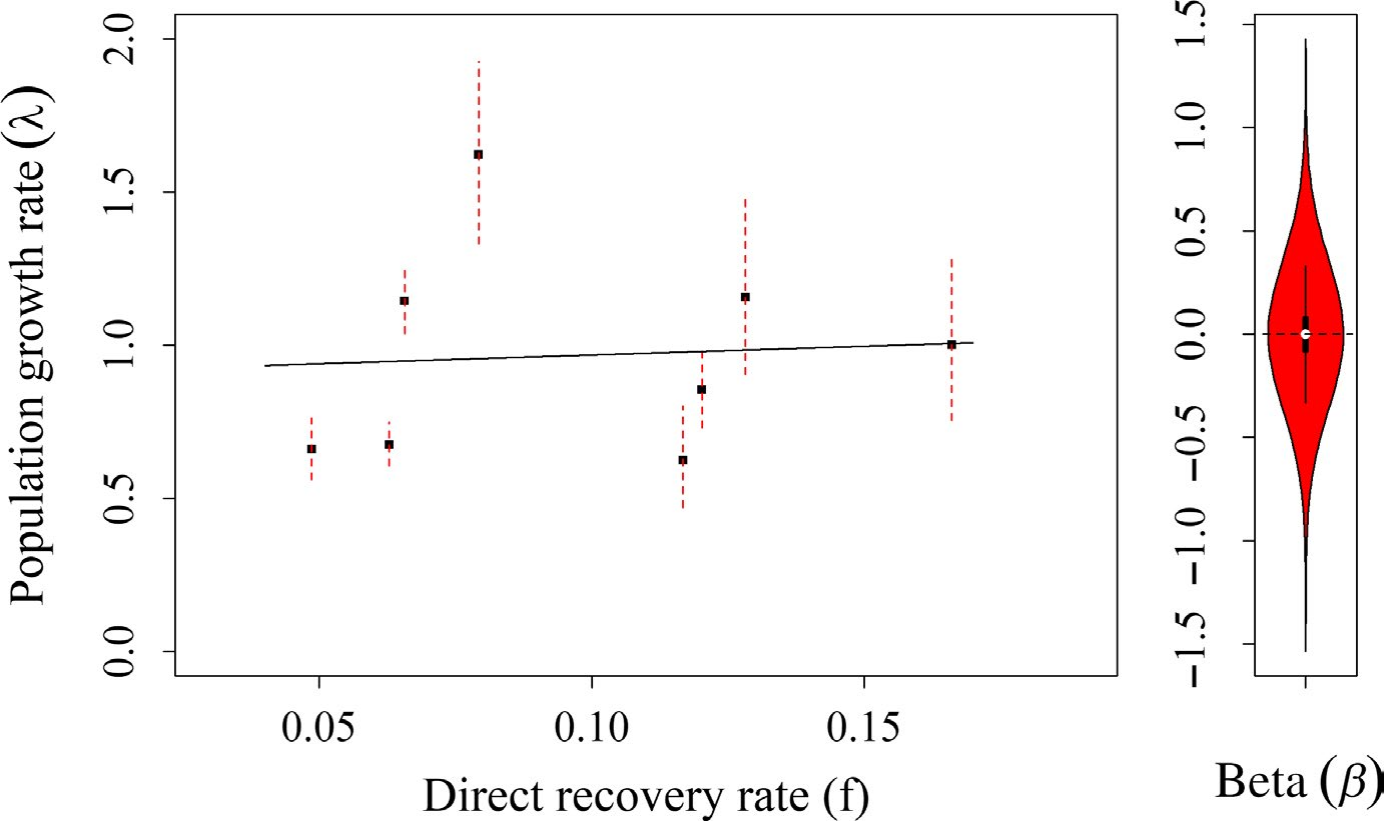
Relationship between the population growth rate (*λ*) and direct recovery rate in the study area from wood duck capture–mark–recapture study in Churchill county, Nevada (2008–2016). Beta represents the slope coefficient from regressions analysis and broadly overlaps zero indicating no effect (*β* = −0.001, 95% BCI: −0.311 to 0.314, *f* = 0.501)

Generally, assessments of harvest effects focus on specific vital rates like survival, but managers are ultimately interested in population‐level effects. Harvest during autumn and winter months did not affect change in the breeding population from 1 year to the next supporting the hypothesis that harvest mortality is being compensated, through reductions in natural mortality, increased recruitment, or both (Figure 6). If harvest was not being compensated for, we would expect it to negatively affect change in the breeding population. Assessing harvest effects on population growth rate (*λ*) provides a relatively straightforward approach to studying population‐level response to harvest, though it does not provide any mechanistic explanation. If our goal is to understand how harvest affects population dynamics, then focusing on specific vital rates will be important. However, from a management perspective, focusing on population‐level effects of harvest is the ultimate goal. Surveys provide a useful index for waterfowl populations, though we believe CMR approaches could provide less biased estimates of abundance while also providing estimates of age and sex ratios that could be used in integrated population models to better inform our understanding or population dynamics (Alisauskas et al., 2014; Arnold, Afton, Anteau, Koons, & Nicolai, 2016).

We believe climate change and habitat loss represent the greatest threats to waterfowl populations in the future (Niemuth, Fleming, & Reynolds, 2014; Reese & Skagen, 2017; Sorenson, Goldberg, Root, & Anderson, 1998). While there are also concerns about harvest effects on waterfowl populations, our results indicate that harvest is not affecting annual change in abundance of breeding wood ducks. Further, there is a growing body of research showing duck harvest is at least partially compensated for in the mortality process, for example, mallard (Anderson & Burnham, 1976), pintail (Bartzen & Dufour, 2017; Rice et al., 2010), redhead (Péron, Nicolai,& Koons, 2012), and lesser scaup (Arnold et al., 2016; 2017), although see Lindberg, Boomer, Schmutz, and Walker (2017) for a cautionary statement. We acknowledge unregulated harvest can be detrimental to duck populations (Bellrose, 1976) but given limited resources available to study and manage wildlife, we question whether an intense focus on managing harvest is benefiting waterfowl populations in the ways intended, especially for ducks. Consequently, we believe monitoring programs should be adjusted to better evaluate the effects of habitat loss and shifts in climatic conditions as we move into an uncertain future.

## CONFLICT OF INTEREST

None declared.

## AUTHOR CONTRIBUTIONS

CAN and RW: designed the study. BSS, CAN, and WH: collected and analyzed data. TVR: assisted with analyses. BSS: wrote the paper with assistance from TVR, KMS, RW, WH, and CAN. All authors contributed to revisions and writing the paper.

## Data Availability

The data are archived in the Dryad Digital Repository: https://doi.org/10.5061/dryad.573n5tb3d.
