# Supplementary material for: Experimental harvest regulations reveal that water availability during spring, not harvest, affects change in a waterfowl population
Source: Ecol Evol. 2019 Oct 28;9(22):12701–9. doi: 10.1002/ece3.5743 (PMC6875577; doi:10.1002/ece3.5743)
Supplement: Supplementary file 1 [file ECE3-9-12701-s001.docx]

Appendix S1. Table of model results from Bayesian closed capture analysis of wood duck capture-mark-recapture data from Churchill County, Nevada (2008-2016). B. Sedinger, T. Riecke, C. Nicolai, R. Woolstenhulm, B. Henry, K. Stewart. Experimental harvest regulations reveal that water availability during spring, not harvest, affects change in a waterfowl population. Ecology & Evolution.

| parameter | mean | sd | q2.5% | q25% | q50% | q75% | q97.5% | Rhat | n.eff | overlap 0 | f |
| --- | --- | --- | --- | --- | --- | --- | --- | --- | --- | --- | --- |
| N.der[1] | 787 | 96 | 628 | 720 | 778 | 843 | 999 | 1.00 | 810 | 0 | 1 |
| N.der[2] | 776 | 52 | 685 | 740 | 773 | 809 | 889 | 1.00 | 9000 | 0 | 1 |
| N.der[3] | 894 | 96 | 736 | 824 | 884 | 951 | 1106 | 1.00 | 9000 | 0 | 1 |
| N.der[4] | 551 | 49 | 468 | 516 | 546 | 581 | 659 | 1.00 | 3113 | 0 | 1 |
| N.der[5] | 887 | 35 | 825 | 863 | 886 | 910 | 961 | 1.00 | 863 | 0 | 1 |
| N.der[6] | 757 | 54 | 663 | 719 | 752 | 790 | 872 | 1.00 | 2268 | 0 | 1 |
| N.der[7] | 497 | 17 | 467 | 485 | 496 | 508 | 534 | 1.00 | 9000 | 0 | 1 |
| N.der[8] | 568 | 20 | 533 | 554 | 567 | 581 | 612 | 1.00 | 9000 | 0 | 1 |
| N.der[9] | 383 | 16 | 355 | 372 | 382 | 393 | 416 | 1.00 | 6340 | 0 | 1 |
| p[1] | 0.2298 | 0.0293 | 0.1756 | 0.2097 | 0.2291 | 0.2488 | 0.2891 | 1.00 | 785 | 0 | 1 |
| p[2] | 0.3501 | 0.0260 | 0.2993 | 0.3325 | 0.3502 | 0.3674 | 0.4010 | 1.00 | 9000 | 0 | 1 |
| p[3] | 0.2470 | 0.0273 | 0.1947 | 0.2282 | 0.2469 | 0.2657 | 0.3011 | 1.00 | 9000 | 0 | 1 |
| p[4] | 0.3329 | 0.0321 | 0.2717 | 0.3109 | 0.3326 | 0.3546 | 0.3967 | 1.00 | 3945 | 0 | 1 |
| p[5] | 0.4652 | 0.0214 | 0.4228 | 0.4508 | 0.4656 | 0.4794 | 0.5075 | 1.00 | 1115 | 0 | 1 |
| p[6] | 0.3435 | 0.0270 | 0.2911 | 0.3250 | 0.3432 | 0.3617 | 0.3969 | 1.00 | 1995 | 0 | 1 |
| p[7] | 0.5666 | 0.0250 | 0.5158 | 0.5504 | 0.5675 | 0.5835 | 0.6140 | 1.00 | 9000 | 0 | 1 |
| p[8] | 0.5452 | 0.0241 | 0.4969 | 0.5288 | 0.5453 | 0.5615 | 0.5920 | 1.00 | 9000 | 0 | 1 |
| p[9] | 0.5547 | 0.0287 | 0.4995 | 0.5356 | 0.5547 | 0.5744 | 0.6103 | 1.00 | 9000 | 0 | 1 |
| omega[1] | 0.3933 | 0.0491 | 0.3107 | 0.3588 | 0.3887 | 0.4228 | 0.5027 | 1.00 | 938 | 0 | 1 |
| omega[2] | 0.3883 | 0.0280 | 0.3385 | 0.3687 | 0.3865 | 0.4059 | 0.4476 | 1.00 | 9000 | 0 | 1 |
| omega[3] | 0.4468 | 0.0492 | 0.3644 | 0.4123 | 0.4421 | 0.4765 | 0.5564 | 1.00 | 9000 | 0 | 1 |
| omega[4] | 0.2757 | 0.0262 | 0.2298 | 0.2575 | 0.2737 | 0.2922 | 0.3324 | 1.00 | 2720 | 0 | 1 |
| omega[5] | 0.4438 | 0.0206 | 0.4051 | 0.4297 | 0.4430 | 0.4574 | 0.4867 | 1.00 | 977 | 0 | 1 |
| omega[6] | 0.3785 | 0.0288 | 0.3262 | 0.3583 | 0.3767 | 0.3967 | 0.4393 | 1.00 | 2332 | 0 | 1 |
| omega[7] | 0.2488 | 0.0129 | 0.2245 | 0.2399 | 0.2485 | 0.2572 | 0.2755 | 1.00 | 9000 | 0 | 1 |
| omega[8] | 0.2843 | 0.0142 | 0.2579 | 0.2744 | 0.2837 | 0.2936 | 0.3132 | 1.00 | 9000 | 0 | 1 |
| omega[9] | 0.1917 | 0.0117 | 0.1697 | 0.1835 | 0.1916 | 0.1994 | 0.2156 | 1.00 | 7061 | 0 | 1 |
| lambda.der[1] | 1.0010 | 0.1376 | 0.7544 | 0.9057 | 0.9936 | 1.0880 | 1.3000 | 1.00 | 955 | 0 | 1 |
| lambda.der[2] | 1.1560 | 0.1456 | 0.9040 | 1.0530 | 1.1450 | 1.2470 | 1.4800 | 1.00 | 9000 | 0 | 1 |
| lambda.der[3] | 0.6234 | 0.0850 | 0.4683 | 0.5642 | 0.6180 | 0.6776 | 0.8023 | 1.00 | 9000 | 0 | 1 |
| lambda.der[4] | 1.6220 | 0.1532 | 1.3310 | 1.5170 | 1.6210 | 1.7270 | 1.9260 | 1.00 | 2203 | 0 | 1 |
| lambda.der[5] | 0.8540 | 0.0690 | 0.7303 | 0.8063 | 0.8501 | 0.8982 | 1.0010 | 1.00 | 9000 | 0 | 1 |
| lambda.der[6] | 0.6604 | 0.0515 | 0.5611 | 0.6253 | 0.6600 | 0.6948 | 0.7623 | 1.00 | 3028 | 0 | 1 |
| lambda.der[7] | 1.1440 | 0.0558 | 1.0380 | 1.1060 | 1.1430 | 1.1820 | 1.2560 | 1.00 | 9000 | 0 | 1 |
| lambda.der[8] | 0.6745 | 0.0363 | 0.6065 | 0.6492 | 0.6732 | 0.6984 | 0.7495 | 1.00 | 9000 | 0 | 1 |
| deviance | 15430 | 242 | 14950 | 15260 | 15430 | 15590 | 15900 | 1 | 1464 | 0 | 1 |
