# Supplementary material for: Experimental harvest regulations reveal that water availability during spring, not harvest, affects change in a waterfowl population
Source: Ecol Evol. 2019 Oct 28;9(22):12701–9. doi: 10.1002/ece3.5743 (PMC6875577; doi:10.1002/ece3.5743)
Supplement: Supplementary file 2 [file ECE3-9-12701-s002.docx]

| parameter | mean | sd | q2.5% | q25% | q50% | q75% | q97.5 | Rhat | n.eff | Overlap 0 | f |
| --- | --- | --- | --- | --- | --- | --- | --- | --- | --- | --- | --- |
| alpha | 0.9648 | 0.1499 | 0.6706 | 0.8873 | 0.9659 | 1.0428 | 1.2547 | 1.00 | 32724 | 0 | 1.00 |
| beta.snow | 0.2559 | 0.1600 | -0.0553 | 0.1729 | 0.2561 | 0.3392 | 0.5700 | 1.00 | 43261 | 1 | 0.96 |
| beta.harvest | -0.0008 | 0.1596 | -0.3129 | -0.0833 | -0.0011 | 0.0818 | 0.3116 | 1.00 | 35073 | 1 | 0.50 |
| sigma | 0.3736 | 0.2055 | 0.1753 | 0.2542 | 0.3224 | 0.4274 | 0.8786 | 1.00 | 3892 | 0 | 1.00 |
| fit | 0.8810 | 1.3302 | 0.3634 | 0.4560 | 0.5914 | 0.8788 | 3.0252 | 1.02 | 51000 | 0 | 1.00 |
| fit.new | 1.4666 | 3.6130 | 0.1405 | 0.4204 | 0.7662 | 1.4697 | 6.8033 | 1.08 | 51000 | 0 | 1.00 |
| bpvalue | 0.5816 | 0.4933 | 0.0000 | 0.0000 | 1.0000 | 1.0000 | 1.0000 | 1.00 | 42117 | 1 | 1.00 |
| residual[1] | 0.0615 | 0.3064 | -0.5335 | -0.0978 | 0.0609 | 0.2210 | 0.6624 | 1.00 | 45444 | 1 | 0.61 |
| residual[2] | 0.0878 | 0.1982 | -0.2924 | -0.0147 | 0.0866 | 0.1882 | 0.4769 | 1.00 | 51000 | 1 | 0.72 |
| residual[3] | -0.4156 | 0.1712 | -0.7475 | -0.5037 | -0.4169 | -0.3284 | -0.0790 | 1.00 | 51000 | 0 | 0.99 |
| residual[4] | 0.1118 | 0.3873 | -0.6392 | -0.0895 | 0.1100 | 0.3124 | 0.8618 | 1.00 | 51000 | 1 | 0.65 |
| residual[5] | 0.0916 | 0.2166 | -0.3323 | -0.0216 | 0.0904 | 0.2052 | 0.5162 | 1.00 | 23933 | 1 | 0.71 |
| residual[6] | -0.2154 | 0.2505 | -0.7008 | -0.3452 | -0.2167 | -0.0873 | 0.2803 | 1.00 | 21736 | 1 | 0.86 |
| residual[7] | 0.3078 | 0.2097 | -0.0978 | 0.1998 | 0.3074 | 0.4151 | 0.7208 | 1.00 | 18873 | 1 | 0.95 |
| residual[8] | -0.0112 | 0.2618 | -0.5191 | -0.1475 | -0.0109 | 0.1234 | 0.5065 | 1.00 | 18163 | 1 | 0.52 |
| predicted[1] | 0.9398 | 0.3064 | 0.3389 | 0.7804 | 0.9405 | 1.0991 | 1.5349 | 1.00 | 45444 | 0 | 0.99 |
| predicted[2] | 1.0685 | 0.1982 | 0.6794 | 0.9681 | 1.0697 | 1.1709 | 1.4487 | 1.00 | 51000 | 0 | 1.00 |
| predicted[3] | 1.0390 | 0.1712 | 0.7024 | 0.9518 | 1.0403 | 1.1271 | 1.3709 | 1.00 | 51000 | 0 | 1.00 |
| predicted[4] | 1.5107 | 0.3873 | 0.7606 | 1.3101 | 1.5124 | 1.7119 | 2.2617 | 1.00 | 51000 | 0 | 1.00 |
| predicted[5] | 0.7624 | 0.2166 | 0.3378 | 0.6488 | 0.7636 | 0.8756 | 1.1863 | 1.00 | 23933 | 0 | 1.00 |
| predicted[6] | 0.8758 | 0.2505 | 0.3801 | 0.7477 | 0.8771 | 1.0056 | 1.3612 | 1.00 | 21736 | 0 | 1.00 |
| predicted[7] | 0.8366 | 0.2097 | 0.4236 | 0.7292 | 0.8370 | 0.9446 | 1.2422 | 1.00 | 18873 | 0 | 1.00 |
| predicted[8] | 0.6857 | 0.2618 | 0.1681 | 0.5511 | 0.6855 | 0.8220 | 1.1937 | 1.00 | 18163 | 0 | 0.99 |
| deviance | 4.3646 | 4.9420 | -1.5995 | 0.8154 | 3.2043 | 6.6305 | 17.0197 | 1.00 | 1964 | 1 | 0.84 |

Appendix S2. Table of model results from Bayesian regression analysis of wood duck capture-mark-recapture data from Churchill County, Nevada (2008-2016). B. Sedinger, T. Riecke, C. Nicolai, R. Woolstenhulm, B. Henry, K. Stewart. Experimental harvest regulations reveal that water availability during spring, not harvest, affects change in a waterfowl population. Ecology & Evolution.
